# Psychopathological consequences and dysfunctional behaviours during the COVID-19 epidemic in italy: A longitudinal study before and after the lockdown

**DOI:** 10.1192/j.eurpsy.2021.282

**Published:** 2021-08-13

**Authors:** E. Cassioli, E. Rossi, G. Castellini, G. Sanfilippo, C. Silvestri, F. Voller, V. Ricca

**Affiliations:** 1 Psychiatry Unit, Department Of Health Sciences, University of Florence, Florence, Italy; 2 Agenzia Regionale di Sanità Toscana, Florence, Italy

**Keywords:** post-traumatic stress disorder, quarantine, COVID-19, Depression

## Abstract

**Introduction:**

In the first months of 2020 the COVID-19 epidemic spread in Italy, and the Italian government implemented a general lockdown. These events are at high risk for psychiatric symptoms in the general population, including anxiety/depression and post-traumatic stress symptoms (PTSS).

**Objectives:**

To characterize the psychopathological correlates of the spread of COVID-19 and lockdown in a sample of subjects from the Italian population, with a before-after follow-up.

**Methods:**

Six weeks after the lockdown, 671 subjects aged 18-60 years completed the Brief Symptom Inventory and Impact of Event Scale-Revised, for the evaluation of psychopathology and PTSS respectively. Environmental factors and subjectively-perceived deteriorations related to COVID-19 were also investigated. Pre-COVID-19 data on psychopathology, collected in December 2019/January 2020, were available for 130 subjects and were used for longitudinal analyses.

**Results:**

With respect to males, female subjects more frequently reported deteriorations of relations (21.5% vs 10.9%), household arguments (26.0% vs 12.6%), sleep quality (47.6% vs 26.6%), episodes of overeating (22.5% vs 12.5%), worries for oneself (19% vs 8.9%) and for loved ones (55.7% vs 36.5%). These changes were associated with increased psychopathology, PTSS, and numerous environmental conditions, including significant economic damage from COVID-19/lockdown. Longitudinal analyses showed an increase in phobic anxiety in the whole sample, and in depression for female subjects only, following the spread of COVID-19. Pre-existing psychopathology was a significant predictor of PTSS.

**Figure:**
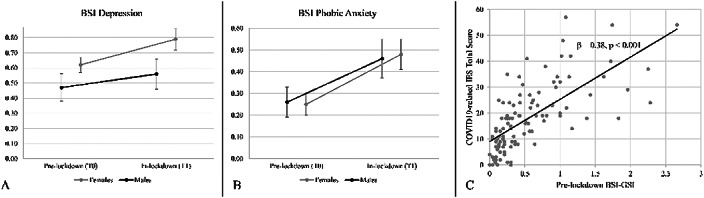

**Conclusions:**

COVID-19 epidemic and lockdown have a high impact on psychopathology and PTSS. Female subjects and those with pre-existing psychopathology were found to be more vulnerable and may need additional support.

**Disclosure:**

No significant relationships.

